# Assessing and Qualifying Neighborhood Walkability for Older Adults: Construction and Initial Testing of a Multivariate Spatial Accessibility Model

**DOI:** 10.3390/ijerph19031808

**Published:** 2022-02-05

**Authors:** Valkiria Amaya, Thibauld Moulaert, Luc Gwiazdzinski, Nicolas Vuillerme

**Affiliations:** 1 AGEIS (Autonomie, Gérontologie, E-santé, Imagerie et Société), Université Grenoble Alpes, 38000 Grenoble, France; nicolas.vuillerme@univ-grenoble-alpes.fr; 2 PACTE (Laboratoire de Sciences Sociales), Sciences Po Grenoble, Université Grenoble Alpes, CNRS, 38000 Grenoble, France; 3 LRA (Laboratoire de Recherche en Architecture), École Nationale Supérieure d’Architecture de Toulouse, Université Fédérale de Toulouse, 31106 Toulouse, France; luc.gwiazdzinski@toulouse.archi.fr; 4 Institut Universitaire de France, 75005 Paris, France

**Keywords:** accessibility, older adults, walking, spatial accessibility model, geographic information systems

## Abstract

Population aging and urban development pose major challenges for societies today. Joining the literature assessing urban accessibility, the present exploratory research developed a multivariate accessibility model based on four independent variables—related to formal and structural urban space—that influence walkability for older adults (pedestrian network; facilities and shops; public benches; and slopes and gradients). The model used ArcGIS software. For the accessibility calculations, we selected two suburban neighborhoods in the conurbation of Grenoble (France) and selected three types of older adults’ profiles to reflect the variety of aging: an older adult in good health, an older adult with a chronic disease, and an older adult with reduced mobility. The results suggest that the accessibility of a neighborhood depends not only on its physical and urban characteristics, but it is also influenced by the physical and health characteristics of its inhabitants. The originality of the model lies mainly in its ability to estimate the spatial accessibility of a territory by taking into account, firstly, objective data such as the physical characteristics and the built environment of the neighborhood through objectification variables that consider such original variables as the presence of benches or the slopes and gradients and, secondly, specific data such as the physical and/or health characteristics of the study population. The measurement of geospatial accessibility could be of great value for public health in urban contexts, which is why relevant tools and methodologies are needed to objectively examine and intervene in public spaces in order to make them age-friendly.

## 1. Introduction

Extended human life expectancy and reduced fertility rates have led to an inevitable change in the population’s age distribution, with a considerable increase in the number of older adults. According to the United Nation’s *World Population Prospects* [1], by 2050, one in six people worldwide (16%) will be over 65 years old, up from one in eleven (9%) in 2019, and the number of people aged 80 or older is expected to triple over the same period. By 2050, one in four people in Europe and North America could be 65 or older. Numerous countries are already looking towards the unprecedented challenges of these expected changes, and new solutions have to be explored today to better meet older adults’ future needs and expectations [2]. 

Firstly, it must be recognized that the different generations within a city have different needs, and that a city’s potential must be exploited for the benefit of all age groups: these two elements are fundamental to making a city friendly to all ages [3]. In this regard, the World Health Organization (WHO)’s 2007 *Age-Friendly Cities* report provides guidelines that could help planners design inclusive neighborhoods and cities.

Recent studies have reported that the physical attributes and characteristics of a neighborhood’s built environment (e.g., access to services and stores, pedestrian-friendly infrastructure, recreational facilities, among others) are factors that promote physical activity levels in older adults [4,5,6,7,8,9], encouraging walking and therefore supporting active and healthy aging [10,11]. Walking is indeed an important part of daily mobility, particularly among the elderly [12]. It hence comes as no surprise that scientists and clinicians [13] but also engineers, policymakers, and urban planners consider walking and mobility in older adults as a major and fruitful focus of study, and numerous reviews on this topic are available (e.g., see for recent reviews [14,15,16]). Accessibility is a major component of this and often highlighted by the WHO [17]. It is commonly considered as implying geographical accessibility to specific places such as outdoor spaces, urban facilities, and services. However, accessibility is also linked to information and social participation. The notion of spatial accessibility was the starting point for the present investigation.

The measurement of accessibility is generally approached in two ways: (i) by attraction to an activity (or opportunities) and (ii) by transport impedance which is calculated based on travel time or distance [18]. There are different methods to address accessibility; however, there are two measures widely used in research [19]. The first is the gravity-based measure, which measures “interaction opportunity potential”, which is affected by opportunity impedance [20], i.e., it discounts the attractiveness of destinations with increasing distances or travel times from the origin. The second is the cumulative opportunity measure, in which accessibility is determined by the number and spatial distribution of activities (or opportunities) within a specified transport impedance [18,21].

Gravity-based [22,23,24,25,26,27,28,29] and cumulative opportunity-based [19,25,30] studies have measured accessibility by investigating various topics, such as accessibility to medical or social services [22,23,30] or to urban services and shops [24,25,26,27,28]. However, these studies [22,23,24,25,26,27,28,29,30] focus on measuring accessibility towards a specific activity or opportunity, i.e., taking into account only one variable of analysis. Few studies [19,31] explore the multidimensional aspects of accessibility or propose a model that seeks a global notion of accessibility. For example, Chen et al. (2020) have measured accessibility for age-restricted communities, considering various types of facilities and services that are important to older adults (i.e., grocery stores, outdoor recreation spaces, health services), in consideration of various influencing factors (i.e., weights and different distance thresholds for these neighboring facilities) [19]. In addition, the physical and health characteristics of the study population are rarely taken into account. It is generally assumed that everyone in a given location has the same level of accessibility, when in reality physical and health limitations affect a person’s ability to access [32]. Furthermore, since research focused on accessibility for older adults is a relatively new field, most studies considered older populations from a macro, regional perspective (e.g., using census or statistical areas) and not at the micro level of their communities [19]. Our study was designed to fill these gaps to guide an interpretation of accessibility through walkability from a comprehensive and multifactorial approach.

This work’s contributions are threefold. 

First, the geographic or spatial accessibility at the neighborhood level (micro level) is calculated from a multivariate perspective, considering various opportunities (variables) that influence the promotion of physical activity and walking in the neighborhood of the older adults [4,33,34,35], such as its pedestrian network system, the locations of services, facilities, and shops, and variables that have been rarely studied but are important in the promotion of walking, such as benches and slopes and gradients.

Second, the study considers the diversity of aging by considering three profiles of older adults: with good health, with a chronic disease, and with reduced mobility. Recent works in social gerontology warn us against the tendency to reduce the identity of older adults to the dimension of age alone, so that more and more work in the social sciences is interested in the problem of heterogeneity and complexity of aging [36].

Third, a multifactorial spatial accessibility model is presented, which considers a diversity of variables related to the physical and urban characteristics of the neighborhood and capable of considering the heterogeneity of the study population based on its physical and health characteristics. The originality of the method presented is characterized by the combination of the severity-based measure and the cumulative opportunity measure, using techniques based on geographic information systems (GIS). This provides a useful reference for studying flexible neighborhood designs that can preserve and promote accessibility and quality of life for older adults.

This paper is organized as follows. Section 2 discusses the study zones, survey methodology, the variables analyzed, dataset construction, and the profiles of different older adult groups. Section 3 presents the accessibility zones by population profile. Section 4 is our discussion, and Section 5 presents our conclusions.

## 2. Methods 

The accessibility model is the result of a set of geospatial analyses following several steps: selection of the study area (Section 2.1); construction and processing of analysis variables (Section 2.2); proximity based on service areas (Section 2.3); map algebra and a weighted sum of variables (Section 2.4); and distinguishing older population profiles (Section 2.4.1). 

Our geospatial analyses used ArcGIS version 10.4 software (Environmental Systems Research Institute (ESRI), Redlands, CA, USA), the Map Algebra tool, and the Network Analyst extension, as is common in many published studies on the topic [22,28,29].

### 2.1. Study Area

**Figure 1:**
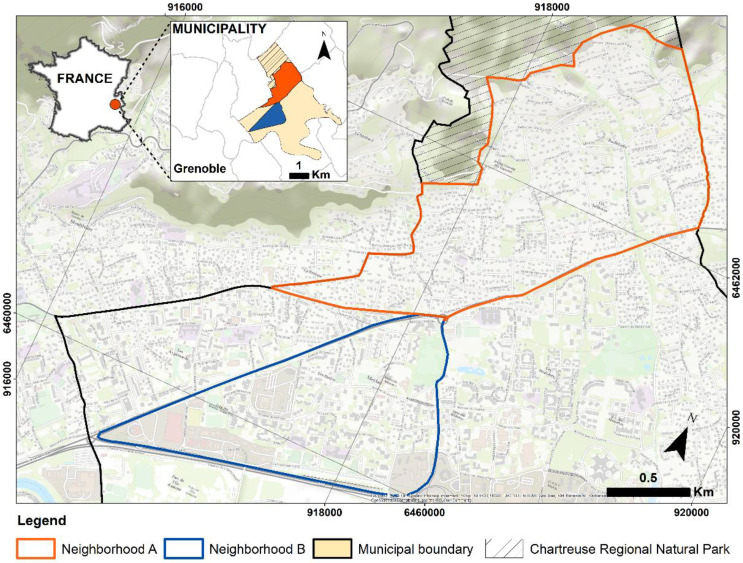
Location of the study areas. Maps created by the authors with ArcGIS 10.4 (https://www.esri.com/en-us/home, accessed on: 23 July 2021). Background image: Esri, OpenStreetMap.

The study took place in a medium-sized town in the Grenoble conurbation in France (Figure 1). This municipality, like many others in France, is affected by the phenomenon of demographic aging [37]. This is a profound and long-lasting trend linked to the increase in longevity and accentuated by the arrival at retirement age of the large baby-boom classes [38]. Characterized as one of the municipalities of the Grenoble urban agglomeration with the highest concentration of people over 60 years of age and with an average growth rate of this population group of 1.5% per year, the municipality has 17,129 inhabitants; people over 60 years of age represent 32.8% of the population of the municipality [39], compared to 21% for the entire urban agglomeration of Grenoble. Our study area included two neighborhoods: A and B, with 34.2% and 36.7% of their inhabitants over 60 years old, respectively. Both were chosen because they represented contrasting urban forms, functional organizations, topography, and urban atmospheres [38]. Our study has been carried out taking into account the optimal climatic and meteorological conditions that favor walkability [40,41,42], conditions that correspond to spring and summer (agreeable temperatures, no rain, no wind, and no heat waves).

Neighborhood A is 3.8 km^2^, covering a large part of a site of the first settlements in the municipality and which, from the 1960s onwards, experienced an accelerated development through residential development of individual housing, characterized by houses with large gardens. We did not consider 1.7 km^2^ of the north of this neighborhood as it is part of the Chartreuse Regional Natural Park.

Neighborhood B is 1.3 km^2^, located at the town’s center and south of Neighborhood A. It experienced the second and third phase of massive urbanization since 1970, characterized by the presence of collective buildings. It is the municipality’s administrative center and is characterized by a dense network of pedestrian and cycling routes, in addition to its functional variety, where housing, commercial activities, and green areas are intermingled.

### 2.2. Analysis Variables

We approached neighborhood accessibility through an analysis of qualitative and quantitative data. To this end, we relied on four variables widely recognized and used in the literature related to the calculation of accessibility indicators [4,5,34,35] as elements that could contribute to and promote walkability for the older adults: (1) the neighborhood’s pedestrian network and its connectivity; (2) the neighborhood’s functional mix of infrastructure (services, facilities, and shops); (3) public benches; and (4) the slopes and gradients encountered when walking between them all.

This section describes the importance of each of the variables considered in the accessibility calculation model.

#### 2.2.1. Data Sources

Concerning road infrastructure, services, and facilities, we used the official “BD TOPO“ (https://geoservices.ign.fr/bdtopo, accessed on 11 August 2020) database of 2018, from the French National Institute of Geographic Information and Forestry (IGN). In addition, we used high resolution aerial photographs (20 cm of spatial resolution) of 2018 from the “BD ORTHO” (https://geoservices.ign.fr/bdortho, accessed on 6 September 2020) IGN database, as a reference for the complete pedestrian network. Furthermore, these databases have been evaluated and updated with in situ observations. Regarding the slopes and gradients, this variable was computed using the digital terrain model (DTM) of 25 m resolution from the “BD ALTI“ (https://geoservices.ign.fr/bdalti, accessed on 11 November 2020) IGN database of 2015. Finally, all these spatial data have been homogenized under the RGF-L93 (EPSG:2154) projection system (official projection for maps of metropolitan France).

#### 2.2.2. Pedestrian Network

**Figure 2:**
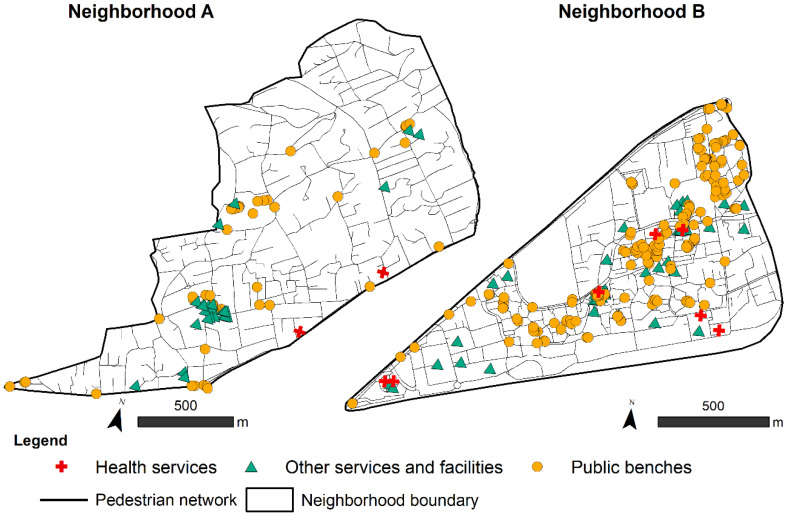
Analysis variables for calculating accessibility by neighborhood. Maps created by the authors with ArcGIS 10.4.

The quality of a pedestrian network, viewed as an aid to travel and mobility, determines the duration of the trip and depends mainly on its morphology, which can be described by the notion of connectivity [7]. To describe connectivity, we collected a dataset including the studied areas’ detailed road networks, provided by the National Institute of Geographic and Forestry Information. These data were used as a reference for constructing each neighborhood’s pedestrian network, considering every path where a pedestrian might walk (sidewalks, footpaths, pedestrian crossings) in a very precise way. This involved several field visits and the collection of complementary aerial photographs of the study neighborhoods. In addition, bicycle paths, which were not included in the road network data, were added to our maps manually, as were roads without sidewalks, which were nevertheless shared by vehicles, bicycles, and pedestrians (a characteristic of some roads in Neighborhood A). In total, we surveyed 45.35 km of pedestrian network in Neighborhood A and 41.07 km in Neighborhood B (Figure 2). 

#### 2.2.3. Services and Facilities

Age-friendly communities seek physical and mental well-being by providing at least three elements: mobility, adequate open spaces, and adequate access to nearby facilities [19]. For the latter element, the most important destinations and facilities for the elderly are medical services, emergency services, municipal facilities, social and cultural facilities, shopping, and entertainment [31].

Based on the selection of services and facilities in two previously published studies [19,31], we added school and childcare facilities such as colleges, schools, and nurseries because they are considered important destinations for older people who are grandparents. Indeed, they represent important functional spaces that encourage older adults to start the physical activity [43] of picking up their grandchildren from school. 

We constructed five groups of services and facilities: (i) health services (including medical and emergency services); (ii) municipal facilities and services; (iii) social and cultural facilities; (iv) shopping; and (v) school and childcare facilities. Details are provided in Table 1.

#### 2.2.4. Urban Furniture: The Case of Public Benches

Since the launch of the WHO program supporting Age-Friendly Cities and Communities, the presence of benches has generally been considered a necessary and legitimate amenity for older people. Many older adults can hardly walk around their neighborhoods without resting on a bench, and several cities, such as New York, have implemented specific programs based on this age-friendly perspective [44]. Extensive research has been conducted to determine the role and importance of benches for the general population [45,46] as well as older adults [34,47,48,49]. As there was no database containing this information, we surveyed all existing public benches, also considering bus stop benches, and some informal seating such as low walls. We considered all these options because one of the key issues in the public bench concept is providing sufficient relay benches, i.e., benches allowing a short break during a walk; if a bus stop bench can be used to wait for a bus, it can also be used as a relay [48,50]. Benches were surveyed using a cell phone application “UrbApp” we have specifically developed for geo-referenced survey of urban furniture. A total of 222 benches were identified across the two neighborhoods (Figure 2): 53 in Neighborhood A and 169 in Neighborhood B. This work was carried out entirely by the first author [51].

#### 2.2.5. Slopes and Gradients

**Figure 3:**
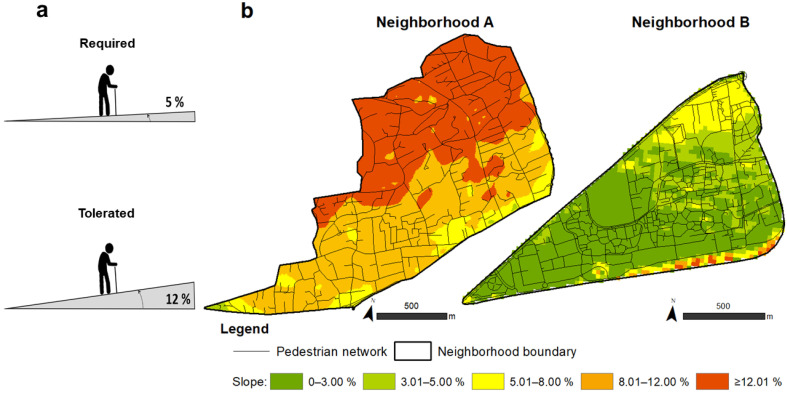
Gradients. (**a**) Representation of the statutory and tolerated gradients according to France’s accessibility regulations. (**b**) Modeling gradients in percent. Maps created by the authors with ArcGIS 10.4.

When walking, the influence of a path’s slope or gradient is crucial to certain pedestrian users’ ability to move, especially elderly pedestrians [8,35,52]. In France, the reference law on accessibility is Law No. 2005-102 of February 11, 2005, “*Law for Equal Rights and Opportunities, Participation, and Citizenship of Persons with Disabilities*” [53]. This law considers an accessible path to be horizontal and without obstacles, and that a path without obstacles is characterized by a gradient equal to or less than 5%. On this basis, we constructed a classification to model the difficulties faced by people walking up and down slopes (Figure 3a). Based on this, we calculated the gradients across both neighborhoods, using a digital terrain model (DTM) with a resolution of 25 m horizontally (Figure 3b). In Neighborhood B’s southeastern sector, an abnormal increase or distortion in gradients can be observed. This was due to the presence of large, tall trees and the artificial elevation of the terrain in this sector that separates the urban area from the highway, elements that generated errors in the DTM.

### 2.3. Proximity Based on Service Areas

GIS distinguishes two components of geographical accessibility [54]: availability, which evaluates the supply, and proximity, which estimates and evaluates the shortest distances. 

**Figure 4:**
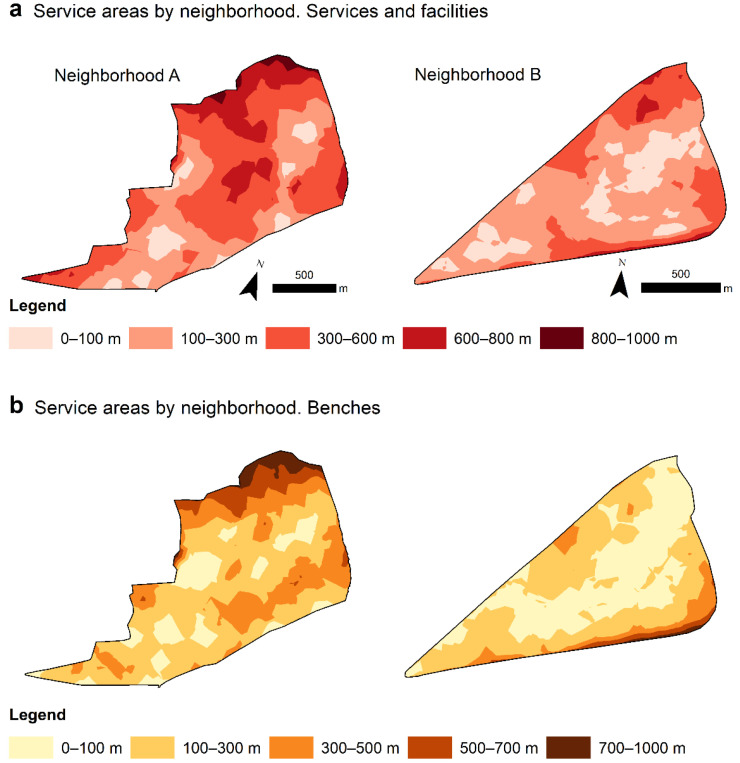
Service areas for “services and facilities” and “benches”. Maps created by the authors with ArcGIS 10.4 and its Network Analyst extension (https://www.esri.com/fr-fr/store/extensions/arcgis-network-analyst, accessed on 23 July 2021).

ArcGIS Network Analyst provides network-based spatial analysis tools. It can be used to plan transportation routes, calculate driving times, and solve other network-related problems. Similarly, it can be used to calculate service areas, which are areas that are within a given distance or can be reached in a specific travel time from a known location [55]. Service areas are commonly used to measure or calculate the accessibility of a certain service or facility by a given transport medium (car, bus, etc.) [29,55,56,57]. However, they can also be applied to walking [24,26]. In our model, therefore, we used the Network Analyst extension to calculate service areas that determined geographical proximity in terms of distance or the effort required of older adults to walk to two of our variables: benches, and services and facilities (Figure 4).

Networks can accumulate large numbers of cost values, such as distance, time, gradient, or other attributes that can influence the cost of travel and walking, including speed. To do this, we based our model of speed profiles by age, sex, and slope developed in the thesis written by V. Nadja [58]. The present study, however, did not consider the sex of older adults’ profiles. We averaged their speeds (4–4.3 km/h for women and 4.4–4.7 km/h for men) and considered just two average speeds: 4.3 km/h where the gradient varied from 0º to 9º (0–15.8%) and 4 km/h where the gradient exceeded 9º (≥15.9%).

### 2.4. Map Algebra to Identify Potentially Accessible Areas

Map algebra constituted our main strategy for obtaining one output layer from the combination of several input layers processed through an algorithm. To compute the accessibility of different neighborhood sectors, we calculated a weighted sum of the layers (representing each of our variables). This process combined a series of steps. First, we homogenized our data format by converting every layer into a regular grid or 5 m raster format (due to the study’s fine scale) and then normalized the criteria by reclassifying the input raster values into a common rating scale. When the input criteria layers used different numbering systems, i.e., with different units (percentages, distances), each cell (pixel) of each criterion had to be reclassified using a common scale. The study of accessibility in urban contexts is a multifaceted topic, and the generality of the term *accessibility* has led to there still being no consensus in the scientific literature on how to qualify it precisely [59]. Our model used five levels of accessibility (very low, low, moderate, high, and very high), levels based on the availability and proximity to a service, commerce, or a bench, and the difficulty of the travel due to slopes and gradients, which allowed us to perform arithmetic operations using values that had originally been of different types (Table 2). Subsequently, each variable (input layer) had to be weighted by its percentage of influence, which was assigned to it according to the characteristics of the profile of the population studied. The choice of weighting criteria for each variable was based on the available literature studying the relationship between neighborhood physical environmental attributes and older adults, which is developed in detail in Section 2.4.1 (e.g., [4,60,61]). Once the weighting criteria were established, and validated collectively by the team, a weighted linear combination or weighted sum analysis was applied, illustrated by Equation (1) [62], in which the cell (pixel) values of each input layer (variable) were multiplied by their respective weighting coefficient and these results were summed to produce a final output layer representing the potential accessibility of the neighborhood.

The weighted sum is illustrated in the following equation:

(1) $$PA=\overset{n}{\underset{i=0}{\sum }}r_{i}\times w_{i}$$

where ***PA*** is the potential accessibility, ***n*** is the total number of variables, ***r_i_*** is the variable or input layer, and ***w_i_*** is the weight assigned to each variable or input layer.

#### 2.4.1. Older Adult Profiles

Although the major contribution of the literature on social gerontology is its emphasis on the diversity of ways of aging, the literature combining quantitative research on living environments and aging [63,64] does not seem to dwell on this. Although some qualitative spatial research methods appear in studies on aging [65], few have compared the perceptions of different groups, such as various ethnic groups or people with disabilities [49,66].

The benefit of distinguishing several population profiles (in our model, three) is taking advantage of both types of approaches, namely quantitative and qualitative. The weighting given to each variable for calculating accessibility according to each of these profiles is, therefore, not necessarily representative of every individual due to our study’s experimental nature (Table 2). It should be noted that the choice of weighting for each variable was based on the available literature where the needs and/or physical and health characteristics of each profile are reported.

##### Profile 1: Healthy Older Adults

Because the presence of nearby services and facilities that promote physical activity among a neighborhood’s older adults represents one of the most important aspects found in the relevant scientific literature [31,33,43], this variable was given a higher weighting (45%) than the others; we considered five groups of services and facilities (Section 2.2.3). We considered that gradients (10% weighting) did not present a disadvantage to healthy older adults, and we assumed that they would have many more leisure and recreational outings, and therefore benches would be a more important variable for them (45% weighting).

##### Profile 2: Older Adults with Chronic Disease (The Case of Diabetes)

Although physical activity is a recognized element in the prevention and management of many chronic diseases associated with aging [43,67], levels of physical activity tend to decrease progressively with age [60,68]. Diabetes among older adults is an increasing worry in Western countries [69]; in France, a quarter of type 2 diabetics are over 75 years old, with an associated lower life expectancy and excess mortality [70]. Therefore, for this group of older adults who need recurrent medical treatment, the presence of nearby health services (health centers, specialized healthcare, and pharmacies) was given greater importance (40% weighting) than other services and facilities (municipal facilities and services, social and cultural facilities, shopping, and school and childcare facilities) (10%), the presence of benches (30%), and the gradients of the neighborhoods (20%).

##### Profile 3: Older Adults with Reduced Mobility Which Use an Assistive Mobility Device (Cane, Walker, and/or Crutches)

As adults age, they may experience a decline in their ability to walk safely, so some use assistive devices such as canes or walkers [71]; therefore, the ability to move around the community safely and easily plays a key role in the lives of people who use mobility devices [49,61]. Although the sense of use of mobility or assistive devices may have a negative effect (ageism) on self-representation as “old” due to others’ views equating it with the limitations of old age [72]. The mobility of these older adults will be strongly influenced by travel conditions [61]; a lower slope correlates with greater overall participation (40% weight), the presence of various public and commercial services in the neighborhood correlates with greater participation in recreation and cultural activities [73] (30% weight), and the presence of resting places (benches) in parks, on trails, and in stores correlates with greater motivation to get out and walk longer distances [41,49] (30%).

## 3. Results

### Accessibility by Older Adult Profile 

**Figure 5:**
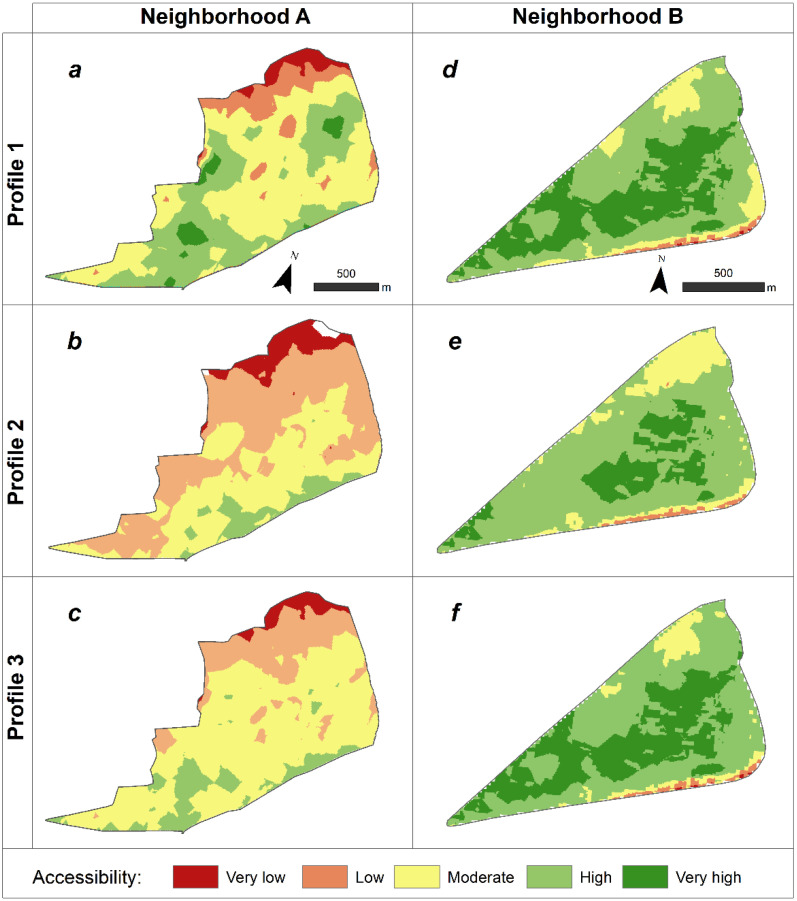
Potential accessibility by neighborhood and by older adult profile: healthy (**a**,**d**), with a chronic disease (**b**,**e**), and with reduced mobility (**c**,**f**), respectively. Maps created by the authors with ArcGIS 10.4.

Figure 5 shows the accessibility sectors for the two older adult profiles in the two neighborhoods studied. Our results showed significant disparities between neighborhoods and their different degrees of accessibility. We observed that levels of accessibility were different across the same neighborhood for the three older adults’ profiles. 

In Neighborhood A, accessibility for healthy older adults (Figure 5a) was generally moderate (across 49% of the area), although we could observe small blocks where accessibility was very high (4%) and high (33%), due to these sectors hosting concentrations of local services and facilities, benches, or good connections to the pedestrian network and moderate gradients (especially in the neighborhood’s southern sector). However, calculations for older adults with a chronic disease profile (Figure 5b) revealed a clearly lower level of accessibility across the neighborhood. We observed high accessibility (7%) in the neighborhood’s southern sector, where two healthcare facilities were located, but in general terms, we observed mostly low (46%) and moderate (39%) accessibility. For Profile 3 older adults with reduced mobility (Figure 5c), we observed moderate accessibility (64%) in most of the neighborhood, small accessible sectors in the south (12%), and low and very low accessibility in the north (24%).

Neighborhood B was much more accessible than Neighborhood A. As explained above, this neighborhood presented with gradients generally less than or equal to 5% (statutory slope), a diversity of services and businesses, a greater bench density than Neighborhood A, and a well-connected pedestrian network system. This neighborhood had no sectors of low accessibility for the three older adult profiles, except for the narrow southeastern sector, which was due to a failure of the DTM (explained in Section 2.2.5).

Regarding accessibility for healthy, Profile 1 older adults (Figure 5d), we calculated very high accessibility in most of the central sector (35%) and high accessibility predominated in most of the neighborhood (52%). For Profile 2 older adults with a chronic disease (Figure 5e), the very accessible sector was significantly smaller (17%) and was also concentrated in the neighborhood’s central sector where medical and health facilities are located. This profile was associated with more moderate-accessibility sectors (15%), particularly in the neighborhood’s northeastern sector, where land gradients gradually increase, exceeding 5% (tolerated slopes). Profile 3, older adults with reduced mobility (Figure 5f), shows very similar levels of accessibility to Profile 1, with high accessibility in general (54%), and high accessibility in the central sector of the neighborhood (35%).

## 4. Discussion

The results illustrated in Figure 5 show different levels of accessibility in both neighborhoods. Even though their proximity (one next to the other), and being part of the same municipality, they present different physical and environmental characteristics, and have been part of different periods of urbanization, which influences the characteristics of the urban environment of each neighborhood. The comparison between both neighborhoods evidences the importance of studying and measuring accessibility at the micro level at smaller scales, such as at the neighborhood, since these are the spaces in which older adults tend to spend a large part of their time [74,75]. Moreover, different levels of accessibility are also observed among the three profiles of older adults studied. It is generally assumed that all people in a given place have the same level of accessibility, when in fact physical and health limitations affect their accessibility levels [33], which suggests the importance of considering the characteristics of the population studied. Although a neighborhood’s accessibility depends on its physical characteristics, and its urban built environment as has been repeatedly demonstrated [24,29,31,76], it also has influences on its inhabitants’ individual characteristics, skills, health status, and physical abilities [32,43]. The quality of urban environments and the health and well-being of their inhabitants are deeply interconnected [77], and physical health, psychological health, and social relationships have been demonstrated to play important roles in older adults’ quality of life [78].

The findings suggest that the accessibility of a neighborhood is not the same for all its inhabitants. An inclusive, age-friendly perspective seems to be an opportunity to renew public action on aging [79]; it implies being particularly aware of the risk of homogenizing the target population’s characteristics instead of considering its diversity. If we wish to refer to a given land area’s accessibility, few widely used quantitative assessment indicators are available.

The present study tried to fill this gap from a spatial, comprehensive, and multifactorial perspective, measuring the geographical accessibility of two neighborhoods by means of our multivariate accessibility model that combines gravity-based and cumulative opportunity measures, i.e., considering proximity and availability. This accessibility model allowed us to test the contribution of new spatial analysis methods for studying how neighborhood environments might affect aging populations. It revealed that many neighborhood characteristics are important to older adults’ lives. Benches proved to be one of these variables. However, a more objective survey might include a category for informal benches (e.g., low walls) and benches at bus stops. This would deserve discussion as part of an investigation into residents’ lived experiences of their neighborhood, rather than a researcher taking charge of a quantitative survey of these benches. Understanding and considering the functions that these types of benches fulfill in a neighborhood could fall under the hypothesis that they function as mobility assistance devices. As a complement to our model, this more qualitative and inductive approach is planned as part of the continuation of the present work.

In terms of limitations, due to the experiential nature of the study, we relied on a series of studies for the selection of variables [19,31,43,47,52] and their weights [31,33,43,60,67,68,69] for the calculation of accessibility zones. However, as mentioned above, it will be important that this point be discussed based on the lived experience in the neighborhood by the residents, as it is the voices of the older adults that need to be more integrated into these processes [80], which is part of our future efforts, since other variables important for the promotion of physical activity outdoors and walking could be included in the analysis and model. For example, the quality of pedestrian routes, which plays an important role for people with mobility problems and high fear of falling [33]; crossings (zebra crossings, footbridges) and road safety signals (e.g., traffic lights), which are necessary to be able to cross streets safely [31]; street furniture such as public toilets and street lighting [47]; naturalistic environments such as gardens and parks and dog-friendly spaces [52]. Furthermore, the study considered accessibility only under optimal spring and summer weather conditions, i.e., agreeable temperatures, no heat waves, no rain, and no wind. However, we are aware that weather and seasonal conditions influence the walking trips of older adults [33,42,81]: e.g., winter, due to icy and snowy conditions and decreased vegetation, is associated with decreased neighborhood participation [82,83]; precipitation is related to puddles and mud on shoulders and dirt roads, which cause slip and fall hazards [81]; heat and sun exposure were also obstacles to getting out walking, especially when there was not enough shade or places to rest [41]. Assessing accessibility during different times of the year can provide valuable information on the role of temporal/seasonal factors on walkability [42,84]. As part of our future efforts, the effects of season and weather on neighborhood accessibility will be considered. Furthermore, a deeper analysis of the diversity of the aging population, considering their demographic characteristics (age and gender), and their physical and health characteristics (e.g., elderly with obesity, with musculoskeletal problems, in wheelchairs, etc.) will be part of future efforts. Recent studies have reported that total walking levels vary little by gender [85,86,87], although there are suggestions that there are consistent gender differences in walking participation for some purposes, including leisure, and that there are gender differences in the impact of age on walking [87]. We therefore believe that further research is needed to improve our understanding of how accessibility and walkability fit into the lives of older women and men.

A more accurate assessment of a neighborhood’s accessibility might also include the analysis of variables in a buffer zone around the study zone because people do not generally limit themselves to accessing necessary goods and services within the administrative or geographical boundaries of where they live. This is known as the *edge effect* [88]. The edge effect is often explained by the fact that services outside the study zone may be more accessible to residents living at its edges. Because these services are not included in the analysis, this may lead to the false impression that these edges exhibit poor accessibility [89].

Despite these limitations, our results themselves highlight some of the study’s strengths. Firstly, to our knowledge, this is the first multifactorial study that examines accessibility from a micro perspective, situated in the neighborhood space, as a place that has an important impact on the health and well-being of older adults [64], from a perspective that is inclusive and aware of the diversity and characteristics of the study population, and furthermore from an approach focused on the walkability of the neighborhood. Secondly, an innovative aspect of the accessibility model is its multivariate approach that includes infrequent and little-studied indicators, such as benches, considered as a necessary and legitimate amenity for older adults [44,45], providing a welcome resting point and facilitating longer trips [49]; and slope, which influences the ability to move [8,35], both indicators as a witness of spatial accessibility. Finally, an important strength is the ability and facility to modulate our model for a diversity of older adults’ profiles, based on the data provided by the literature. Nevertheless, additional validation, involving the participation of older adults themselves in a discussion about the results, would be most valuable and is part of our immediate plans.

## 5. Conclusions

In the most walkable cities, activity is higher throughout the day and throughout the week, across all age and gender groups [90]. Physical and environmental settings that allow older adults to increase their ability to walk safely in their neighborhood result in greater community participation [3]. This is why developing accessibility will continue to be one of the important themes in discussions about promoting healthy and active aging in the coming decades. It is therefore important that researchers develop methods and tools to study and promote age-friendly cities. 

The accessibility model presented here seems to be a relevant tool and could also serve to support original and innovative methodological experiments in the field of aging. The flexibility of this model advantageously enables the measurement of accessibility down to a very fine spatial scale since it can consider and be applied to all the important and relevant components of the area being studied, including the characteristics of the study population and its subgroups. The challenges faced by older adults as pedestrians have moved higher on the agenda in both policy and research at the European level. However, in actual planning, there is still much to be done to facilitate mobility and accessibility for older people [80]. The present findings provide a framework for accessibility analysis. Policymakers and urban planners should be aware that accessibility is sensitive as it is conditioned not only by the environmental and urban factors of the territory, but also influenced by the physical and health characteristics of the study population. Accessibility measurement practices generally do not take “people” into account and continue to maintain the place-based approach [91], so it would be important for urban planners and policymakers to consider this aspect for the development of city improvement policies and the promotion of more age-friendly environments.

## Figures and Tables

**Table 1:** Neighborhood services and facilities.

DETAIL		Neighborhood A (Quantity)	Neighborhood B (Quantity)
Health services	Health centers	0	1
	Specialized health facilities (dental clinic, podiatrist, optician, etc.)	1	4
	Pharmacies	1	2
	Emergency services	0	0
Municipal services and amenities	Gendarmerie	0	2
	Cemetery	1	1
	Post office	1	1
	Townhall	0	1
	Community Center for Social Action (CCAS)	0	1
	Neighborhood house	1	1
Social and cultural facilities	Places of worship	4	0
	Sports facilities	1	2
	Neighborhood meeting hall	2	0
	Retirement home/ nursing home	0	2
	Conservatory	0	1
	Library	1	1
Shopping	Local shops (hairdresser, cheese shop, grocery store, butchers, etc.)	8	9
	Restaurants	1	5
	Banks	0	2
	Shopping center	0	1
	Supermarkets	1	2
	Bars	0	1
Schools and childcare facilities	Schools, nurseries	4	5
Total		27	45

Note: The official 2018 “BD TOPO” database from the French National Institute for Geographic and Forestry Information (IGN) was used for the preparation of the table, which has been updated with in situ observations.

**Table 2:** Weightings for calculating accessibility levels for each older adult profile.

100% Weighting			Variable	Rank (m)	Accessibility Level
Profile 1	Profile 2	Profile 3			
45	30	30	BENCHES	0–100	Very High
				100–300	High
				300–500	Moderate
				500–700	Low
				700–1000	Very Low
45	40	30	* HEALTH SERVICES	0–100	Very High
				100–300	High
				300–600	Moderate
				600–800	Low
				800–1000	Very Low
	10		OTHER SERVICES AND FACILITIES	0–100	Very High
				100–300	High
				300–600	Moderate
				600–800	Low
				800–1000	Very Low
10	20	40	SLOPE GRADIENTS (%)	0–3.00%	Very High
				3.01–5.00%	High
				5.01–8.00%	Moderate
				8.01–12.00%	Low
				≥12.01%	Very Low

Note: Rank corresponds to the values (meters and percentages) determined for the accessibility calculations for each variable. * Services and facilities, for the calculation of Profile 2, were divided into health services (health centers, specialized health facilities, and pharmacies) and other services and facilities (see Table 1).

## Data Availability

The data that support the findings of this study are not publicly available. Data are, however, available from the authors upon reasonable request.
